# Severe co-infection with influenza A virus H3N2 and community-acquired methicillin-susceptible *Staphylococcus aureus* in a child presenting with septic shock, acute respiratory distress syndrome, and necrotizing pneumonia: a rare case report

**DOI:** 10.3389/fcimb.2026.1941945

**Published:** 2026-08-19

**Authors:** Zhangyan Guo, Haifeng Qi, Qian Zhang, Yi Wang, Yanqiang Du

**Affiliations:** Department of Pediatric Intensive Care Unit of Xi’an Children’s Hospital, National Children’s Regional Medical Center (Northwest), Children’s Hospital of Xi’an Jiaotong University, Xi’an, China

**Keywords:** case report, extracorporeal membrane oxygenation, influenza, necrotizing pneumonia, pediatrics, *Staphylococcus aureus*

## Abstract

**Background:**

Influenza co-infection with *Staphylococcus aureus* (*S. aureus*) can cause rapidly fatal necrotizing pneumonia, septic shock, and acute respiratory distress syndrome (ARDS) in children. Although methicillin-resistant *S. aureus* is often highlighted, community-acquired methicillin-susceptible *S. aureus* (CA-MSSA) can also produce equally severe disease.

**Case presentation:**

We report an 8-year-4-month-old male with influenza A (H3N2) who developed septic shock and refractory hypoxemia, requiring immediate intubation. Due to persisting respiratory failure despite maximal ventilation, veno-venous extracorporeal membrane oxygenation (VV-ECMO) was initiated on day 1. Metagenomic next-generation sequencing identified *S. aureus* as the dominant pathogen, and bronchoalveolar lavage fluid culture later confirmed MSSA. After vancomycin failed clinically, the regimen was switched to linezolid. However, on day 15 of linezolid therapy, the patient developed severe linezolid-induced lactic acidosis (LILA), which resolved within 3 days of stopping the drug. The clinical course was further complicated by pneumothorax and multidrug-resistant organism superinfections. After 54 days of intensive care, the patient was discharged in good condition.

**Conclusion:**

This case underscores that during influenza seasons, early empirical anti-staphylococcal therapy should be considered in children with rapidly progressive pneumonia and shock, even when CA-MSSA is suspected. Additionally, routine lactate monitoring is critical during linezolid therapy to enable prompt recognition and management of life-threatening LILA.

## 1 Introduction

Influenza is an acute viral respiratory disease caused by infection of the respiratory tract with influenza viruses that circulate among people worldwide. According to the World Health Organization (WHO), influenza affects approximately 1 billion people annually, resulting in 3 to 5 million severe cases and 290,000 to 650,000 deaths worldwide (World Health Organization, 2019). Secondary bacterial pneumonia is one of the leading causes of disease deterioration and mortality, with *Streptococcus pneumoniae (S. pneumoniae)*, *Streptococcus pyogenes* (S. *pyogenes*), and *Staphylococcus aureus* (S. *aureus*) being the most common pathogens (Bartley et al., 2022). Co-infection with *S. aureus* pneumonia can lead to severe necrotizing pneumonia (NP), with a case-fatality rate as high as 90%, and has been identified as an independent risk factor for influenza-associated mortality in both adults and children (Randolph et al., 2019). Both community-acquired methicillin-susceptible *S. aureus* (CA-MSSA) and community-acquired methicillin-resistant *S. aureus* (CA-MRSA) co-infection with influenza virus can result in NP (Park et al., 2015; Takahashi and Nakamura, 2018; Han et al., 2022; Hu et al., 2024; Wang et al., 2024). However, influenza virus co-infection with *S. aureus* leading to concurrent NP and *S. aureus* detected in blood by mNGS is clinically rare (Bai et al., 2020; Ding et al., 2025). Herein, we report a case of NP and *S. aureus* detected in blood by mNGS caused by co-infection with influenza A virus and CA-MSSA in a pediatric patient. The clinical course and management of this patient are detailed below.

## 2 Case presentation

**Figure 1:**
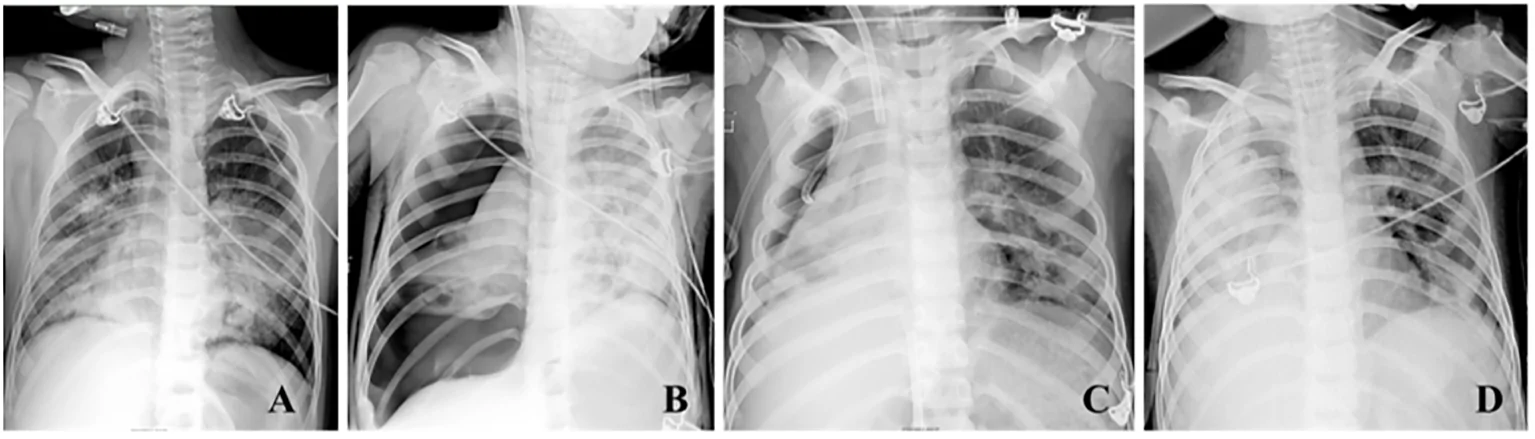
X-ray of the patient. **(A)** Bilateral patchy opacities, suggestive of pneumonia and potential pulmonary hemorrhage (day of admission). **(B)** Right-sided pneumothorax and bilateral patchy opacities with interval worsening (the 10th day of admission). **(C)** Right-sided pneumothorax showed partial resolution, with the appearance of a right pleural effusion; the left-sided patchy opacities had diminished, whereas the number of air-containing cystic lesions had increased (the 29th day of admission). **(D)** Right lung exhibited decreased aeration with patchy densities and cystic lesions; the left lung demonstrated increased and blurred bronchovascular markings with patchy infiltrates, which had progressed locally, while the air-containing cystic lesions remained largely unchanged (the 38th day of admission).

A previously healthy 8-year-4-month-old male was admitted to our hospital on December 9, 2025, with a 12-hour history of fever and a 5-hour history of progressive respiratory distress. He presented to the emergency department (ED) with high fever (peak 40.5 °C), headache, fatigue, and cyanosis. Initial peripheral oxygen saturation (SpO_2_) was 77% in room air, with evidence of bloody secretions in the oropharynx. Given the combination of severe hypoxemia, respiratory distress, and concern for airway compromise from suspected pulmonary hemorrhage, he was immediately intubated. After rapid sequence induction with midazolam (0.2 mg/kg), the patient was successfully intubated with a 6.0-mm cuffed endotracheal tube under direct laryngoscopy. Correct endotracheal tube placement was confirmed by auscultation of bilateral breath sounds and subsequent chest X-ray (CXR), which revealed bilateral patchy opacities suggestive of pneumonia and potential pulmonary hemorrhage (**Figure 1A**). Bloody secretions were visualized within the endotracheal tube, raising the suspicion of pulmonary hemorrhage. Following intubation in the ED, the patient was transferred to the Pediatric Intensive Care Unit (PICU).

Upon PICU admission, his vital signs were as follows: body temperature, 38.2 °C (following antipyretic administration in the ED); heart rate, 162 beats per minute; respiratory rate, 40 breaths per minute; and blood pressure 64/33 mm Hg (1 mmHg = 0.133 kPa). Physical examination revealed central and peripheral cyanosis, coarse breath sounds with bilateral crackles on auscultation, cold extremities with mottling, and a capillary refill time of 5 seconds. Laboratory examination on PICU admission: white blood cell count (WBC) 0.7 × 10^9^/L (reference range: 4.3 × 10^9^/L–11.3 × 10^9^/L), neutrophil (NE) count 0.2 × 10^9^/L (reference range: 1.6 × 10^9^/L–7.8 × 10^9^/L), hemoglobin (HGB) 95 g/L, platelet count (PLT) 100 × 10^9^/L (reference range: 100 × 10^9^/L–300 × 10^9^/L), C-reactive protein (CRP) 104 mg/L (reference range: 0 mg/L–3 mg/L), procalcitonin (PCT) 50.8 ng/mL (reference range: 0 ng/mL–0.05 ng/mL). Liver and renal function tests were normal. A nasopharyngeal swab was positive for influenza A virus (H3N2). Cytokines were obtained on PICU admission due to the severity of the presentation (septic shock); in our practice, cytokine testing is not routine but is performed in patients with septic shock to assess the inflammatory response and guide management: interleukin-1β (IL-1β) 204.7 pg/ml (reference range: 0 pg/mL–5.4 pg/mL), IL-2 48.4 pg/mL (reference range 0 pg/mL–7.5 pg/mL), IL-6 >10,000 pg/mL (reference range 0 pg/mL–5.4 pg/mL), IL-8 5,290.8 pg/mL (reference range 0 pg/mL–20.6 pg/mL), IL-10 1,287.6 pg/mL (reference range 0 pg/mL–12.9 pg/mL), IL-17 102.9 pg/mL (reference range 0 pg/mL–21.4 pg/mL), and interferon-γ (IFN-γ) 53 pg/mL (reference range 0 pg/mL–23.1 pg/mL); tumor necrosis factor-α (TNF-α), IL-4, IL-5, IL-12p70, and IFN-α were normal. The patient was diagnosed with septic shock, severe pneumonia, acute respiratory failure, influenza A infection, and suspected pulmonary hemorrhage.

**Figure 2:**
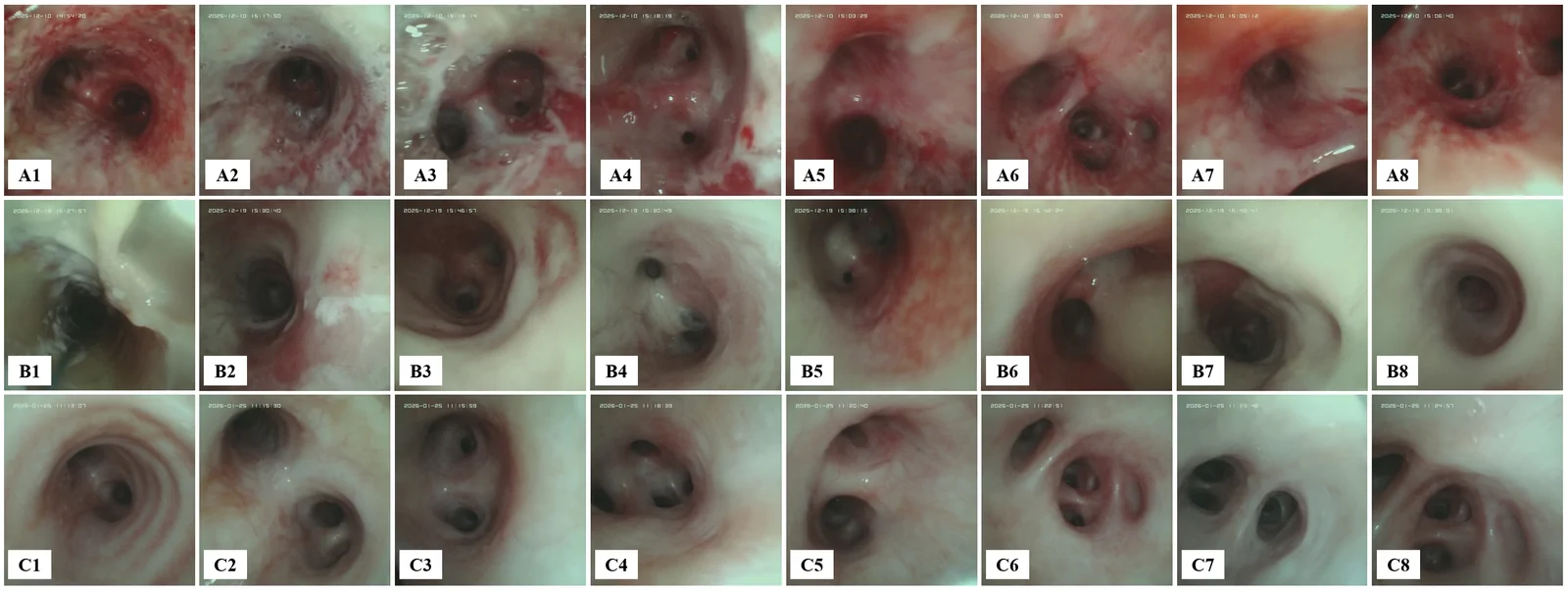
Bronchoscopic findings of the patient. **(A1–A8)** Extensive mucosal erosion and hemorrhagic oozing in the trachea and bilateral bronchi, with abundant yellowish-white secretions in the left lung (the 2nd day of admission). **(B1–B8)** Extensive yellowish-white pseudomembranous formation on the surface of the endotracheal tube, trachea, and left and right main bronchi (the 9th day of admission). **(C1–C8)** The tracheal and bronchial mucosa returned to normal (the 48th day of admission).

**Figure 3:**
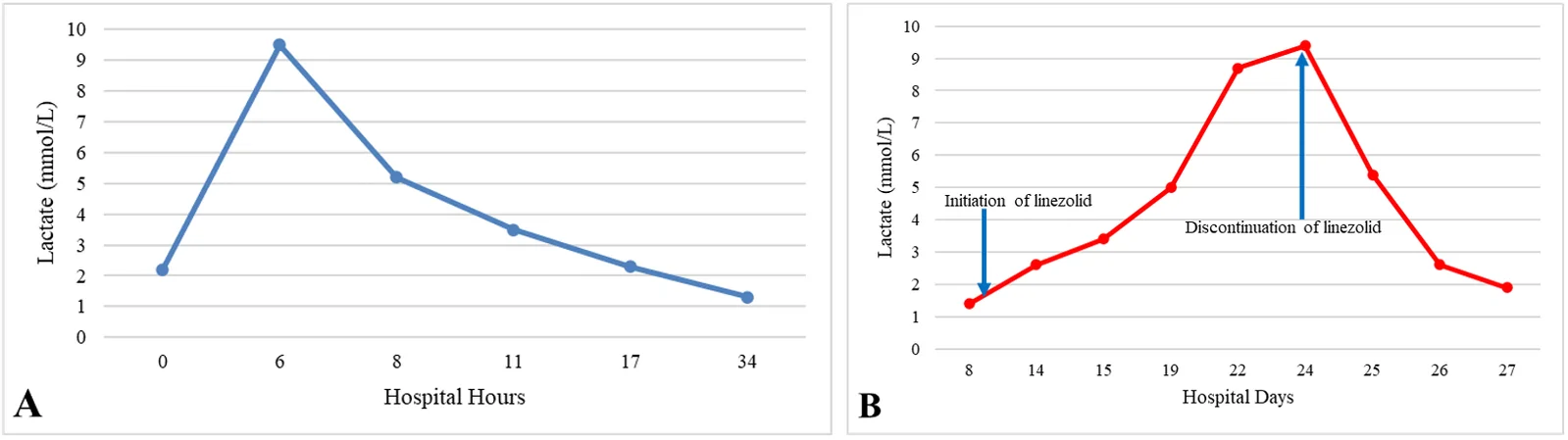
Lactate level of the patient. **(A)** Lactate level progressively increased after admission and then gradually returned to normal. **(B)** After the patient was started on linezolid (the 9th day of admission), lactate levels gradually increased, peaked on day 15 of therapy (the 24th day of admission), and returned to normal three days after discontinuation of the drug (the 27th day of admission).

Initial management included invasive mechanical ventilation (Ventilator parameters: FiO_2_ 1.0, PEEP 10 cmH_2_O, VT 6ml/kg), and vasopressor support with norepinephrine. Empirical antimicrobial therapy was initiated with meropenem, vancomycin, and peramivir. Despite maximal ventilatory support, an arterial blood gas analysis six hours after admission showed: pH 7.35, pCO_2_ 26mmHg, pO_2_ 28mmHg, BE- 10.6mmol/L, lactate 9.5mmol/L, SaO_2_ 51.3%. Owing to refractory hypoxemia, despite FiO_2_ 1.0 and PEEP 10 cmH_2_O, the patient was diagnosed with acute respiratory distress syndrome (ARDS), and veno-venous extracorporeal membrane oxygenation (VV-ECMO) was subsequently initiated on hospital day 1, via percutaneous cannulation of the right femoral vein (15 Fr return cannula) and right internal jugular vein (15 Fr drainage cannula). Continuous renal replacement therapy (CRRT) was initiated for septic shock with severe metabolic acidosis, hyperlactatemia, and markedly elevated cytokines (including IL-6 >10,000 pg/mL) to remove inflammatory mediators; the CRRT circuit was connected in series to the ECMO circuit. A subsequent fiberoptic bronchoscopy revealed extensive tracheobronchial mucosal erosion and scant sanguineous secretions (**Figures 2A1–A8**). The patient’s serum lactate level normalized by 34 hours after admission (**Figure 3A**).

On hospital day 3, metagenomic next generation sequencing (mNGS) of blood detected *S. aureus* (367 reads), *Haemophilus influenzae* (*H. influenzae*) (274 reads), and *S. pyogenes* (45 reads); mNGS of bronchoalveolar lavage fluid (BALF) detected *S. aureus* (57,782 reads), *H. influenzae* (991 reads), *Stenotrophomonas maltophilia* (*S. maltophilia*) (27 reads), and *S. pyogenes* (99 reads). The blood sample and BALF sample were collected on hospital day 2 (approximately 36 hours after antibiotic initiation). mNGS was performed using the Illumina NextSeq 550 platform. Positive results were defined as ≥ 10 reads for pathogenic bacteria and viruses, after excluding background contaminants through internal quality controls and bioinformatic filtering. The Q-index (pathogen index) was used for quantification; in blood, *S. aureus* had a Q-index of 20,605 (ranking above all comparable specimens), and in BALF, it was 20,760 (ranking above 85% of comparable specimens), indicating high microbial loads. Although these additional organisms were detected, the high read abundance of *S. aureus* in BALF, along with the next clinical presentation of NP and pseudomembranous tracheobronchitis, suggested that *S. aureus* was the dominant pathogen. The additional organisms were considered secondary pathogens. Based on these findings, trimethoprim-sulfamethoxazole (TMP-SMZ) was added. On hospital day 4, the patient was weaned off norepinephrine, and hemodynamic stability was achieved. The BALF bacterial culture confirmed methicillin-susceptible *S. aureus* (MSSA); the blood bacterial culture was negative. Identification and antimicrobial susceptibility testing of the MSSA isolate were performed using the bioMérieux VITEK^®^ 2 Compact system with the GP67 card, and minimal inhibitory concentrations (MICs) were determined by the broth microdilution method. Interpretation followed the Clinical and Laboratory Standards Institute (CLSI) M100 guidelines (2025 edition). The oxacillin MIC was 0.5 μg/mL (susceptible: ≤ 2 μg/mL), and the oxacillin screen test was negative, confirming MSSA. The isolate was also susceptible to linezolid (LZD) (MIC 2 μg/mL; susceptible: ≤ 4 μg/mL), vancomycin (MIC ≤ 0.5 μg/mL), and trimethoprim-sulfamethoxazole, but resistant to penicillin and erythromycin. Inducible clindamycin resistance was positive (D-test positive), precluding the use of clindamycin. However, the patient continued to have recurrent fevers (up to 40.0 °C), prompting the continuation of meropenem, vancomycin, and TMP-SMZ.

On hospital day 9, despite ongoing therapy, fever persisted (39.0 °C), and a follow-up bronchoscopy revealed extensive yellowish-white pseudomembranous formation on the surface of the endotracheal tube, trachea, and left and right main bronchi (**Figures 2B1–B8**). Vancomycin was replaced with linezolid (LZD). The switch from vancomycin to LZD was based on multiple considerations: (1) the MSSA isolate was confirmed susceptible to LZD; (2) clinical failure of vancomycin was suggested by persistent fever after 9 days of therapy despite *in vitro* susceptibility to vancomycin; (3) bronchoscopic findings of extensive pseudomembranous formation indicated severe toxin-mediated airway injury, against which LZD—as a protein synthesis inhibitor—may suppress toxin production, whereas vancomycin could potentially promote toxin release; (4) LZD achieves superior pulmonary tissue penetration, which is particularly advantageous in NP with extensive airway involvement; (5) LZD has better activity against *S. aureus* biofilms, which are commonly associated with pseudomembranous tracheobronchitis; and (6) the presence of inducible clindamycin resistance precluded the use of clindamycin as an alternative toxin-suppressive agent.

**Figure 4:**
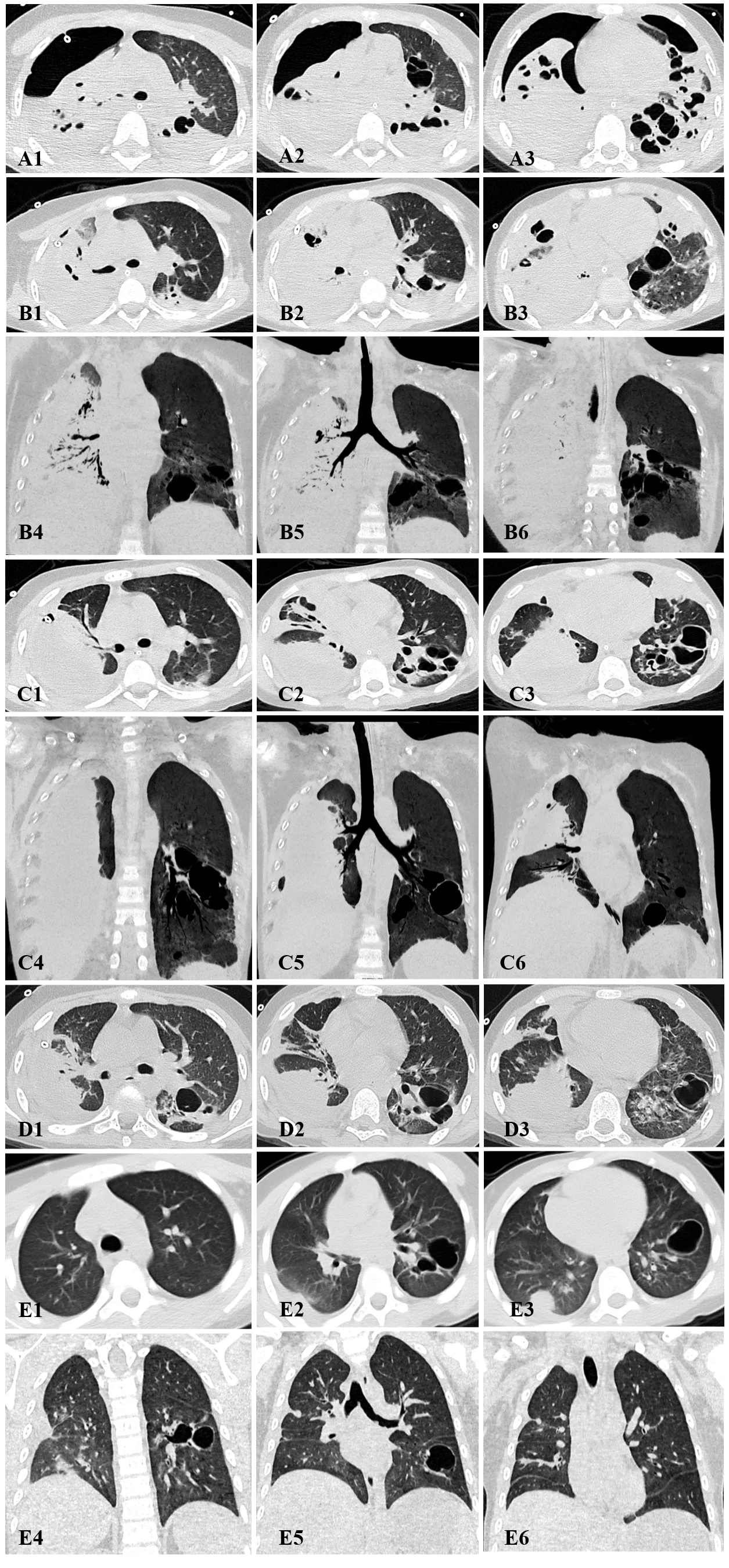
Chest computed tomography (CT) of the patient. **(A1–A3)** Bilateral pneumonia with multiple cavitary lesions and bilateral hydropneumothorax (the 17th day of admission). **(B1–B6)** The left-sided pneumonia had partially resolved, with a reduction in the number of cystic lesions in the left lower lobe, although some had increased in size; bilateral pleural effusions were present, and the bilateral pneumothorax had markedly diminished (the 30th day of admission). **(C1–C6)** The bilateral pneumonia had regressed, with a decrease in the number of cavities; right-sided hydropneumothorax was observed, and the left-sided pleural effusion had largely resolved (the 42nd day of admission). **(D1–D3)** The bilateral pneumonia showed partial resolution, with a reduction in the size of the multiple cavities (the 50th day of admission). **(E1–E6)** The bilateral pneumonia had significantly improved, with the multiple cavities in the left lung being reduced in both number and size (the 38th day after discharge).

On hospital day 10, the clinical course was complicated by a right-sided pneumothorax, necessitating chest tube placement (**Figure 1B**). On hospital day 13, BALF cultures grew carbapenem-resistant *Acinetobacter baumannii* (CRAB) and *S. maltophilia*. Consequently, meropenem was discontinued, and the regimen was changed to cefoperazone-sulbactam, tigecycline, LZD, and TMP-SMZ. On hospital day 14 (day 5 of LZD administration), the serum lactate was 2.6 mmol/L. On hospital day 17, chest computed tomography (CT) showed bilateral pneumonia with multiple cavities, a cystic lesion in the left lower lobe, and bilateral hydropneumothorax (**Figures 4A1–A3**), leading to a diagnosis of NP. On hospital day 19 (day 10 of LZD administration), the serum lactate rose to 5.0 mmol/L. Subsequently, the lactate levels progressively increased, accompanied by recurrent vomiting upon nasogastric feeding, although no gastrointestinal pathology was identified upon screening. On hospital day 24 (day 15 of LZD administration), the serum lactate peaked at 9.4 mmol/L. A diagnosis of linezolid-induced lactic acidosis (LILA) was established, and the drug was discontinued. The patient continued to receive CRRT and was started on oral vitamin B1. On hospital day 27 (day 3 after LZD was discontinued), the serum lactate normalized (**Figure 3B**), and the patient had no further episodes of vomiting.

**Figure 5:**
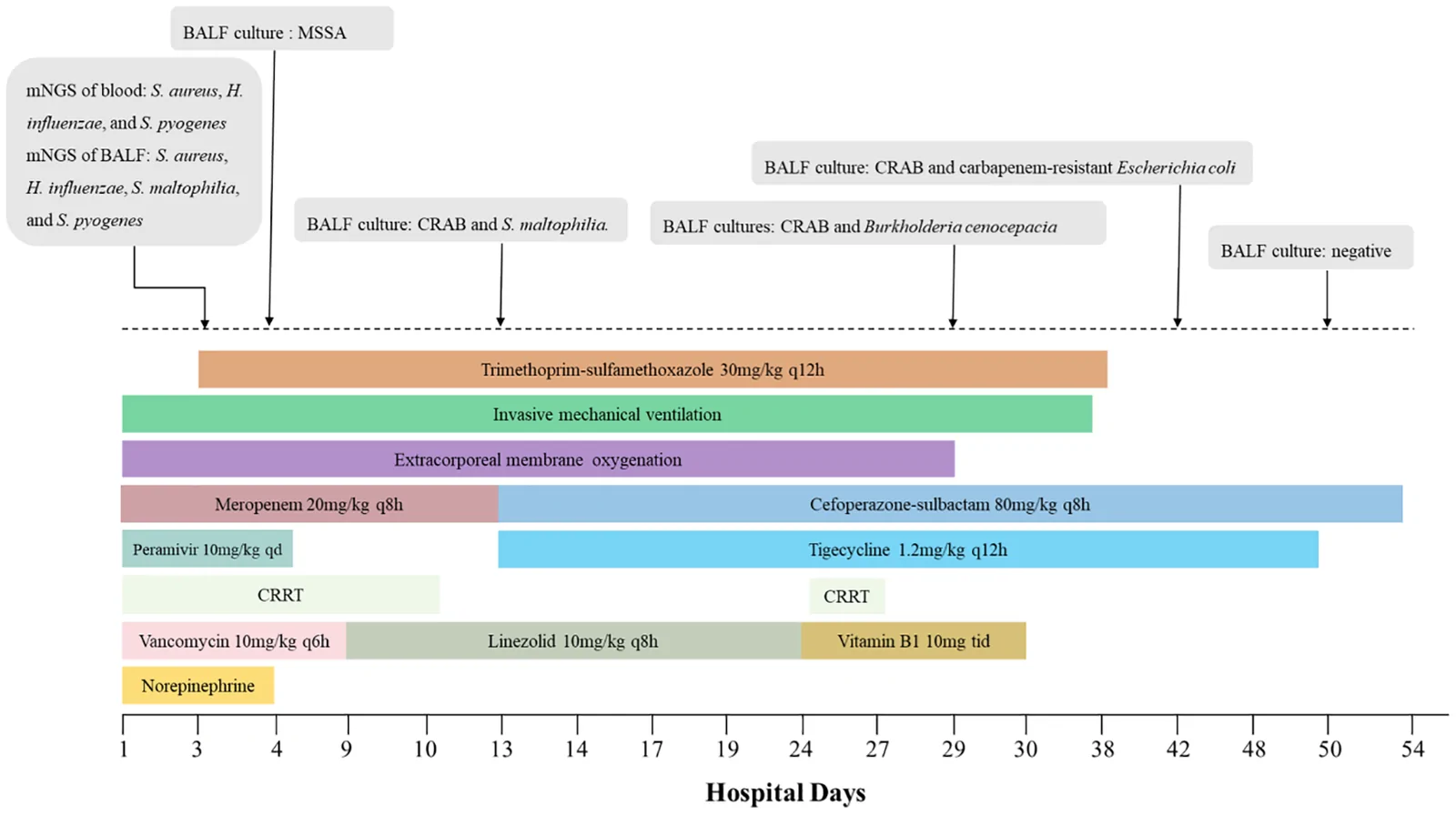
The clinical tests and treatment timeline of the patient. mNGS, metagenomic next generation sequencing; BALF, bronchoalveolar lavage fluid; *S. aureus*, *Staphylococcus aureus*; *H. influenzae*, *Haemophilus influenzae*; *S. maltophilia*, *Stenotrophomonas maltophilia*; and *S. pyogenes*, *Streptococcus pyogenes*; CRAB, carbapenem-resistant *Acinetobacter baumannii*; MSSA, methicillin-susceptible *Staphylococcus aureus*; CRRT, continuous renal replacement therapy.

On hospital day 29, CXR revealed partial resolution of the right-sided pneumothorax, accompanied by a right pleural effusion; the left-sided patchy opacities had decreased, whereas the air-containing cystic lesions had increased in number (**Figure 1C**). Concurrently, the patient was successfully weaned from VV-ECMO on hospital day 29 (after 29 days of support). BALF cultures grew CRAB and *Burkholderia cenocepacia*, and the antibiotic regimen was refined to tigecycline, cefoperazone-sulbactam, and TMP-SMZ. On hospital day 30, a follow-up chest CT was performed, which showed partial resolution of the left-sided pneumonia. The number of cystic lesions in the left lower lobe had decreased, although some had increased in size. Bilateral pleural effusions were also noted, and the bilateral pneumothorax had markedly improved compared with prior imaging (**Figures 4B1–B6**). On hospital day 38, with good spontaneous breathing and a stable CXR (**Figure 1D**), the patient was extubated, and TMP-SMZ was stopped. On hospital day 42, BALF cultures grew CRAB and carbapenem-resistant *Escherichia coli*. Follow-up chest CT revealed regression of the bilateral pulmonary infiltrates, accompanied by a decreased number of cavitary lesions. Additionally, a right-sided hydropneumothorax was present, while the left-sided pleural effusion had nearly resolved (**Figures 4C1–C6**). The regimen was simplified to cefoperazone-sulbactam and tigecycline. On hospital day 48, bronchoscopy revealed the tracheal and bronchial mucosa returned to normal (**Figures 2C1–C8**). On hospital day 50, BALF cultures were negative, and a chest CT demonstrated significant improvement of the pneumonitis and a reduction in the size of the pulmonary cavities (**Figures 4D1–D3**). Tigecycline was subsequently discontinued. On hospital day 54, the patient was discharged home in good condition with no oxygen requirement. On day 38 after discharge, the follow-up chest CT revealed notable regression of the prior pulmonary lesions (**Figures 4E1–E6**). Clinically, the child exhibited satisfactory spontaneous breathing without respiratory distress, and demonstrated good overall mental status and activity tolerance. The clinical tests and treatment timeline of the patient are shown in **Figure 5**.

## 3 Discussion

A Chinese multicenter retrospective study demonstrated that the most common pathogens causing NP in children included *Mycoplasma pneumoniae* (*M. pneumoniae*), *S. pneumoniae*, *S. aureus*, *Pseudomonas aeruginosa*, and *H. influenzae*, with *M. pneumoniae* accounting for 68% of cases (Zhou et al., 2021). However, epidemiological data on influenza virus and *S. aureus* co-infection in children were unknown. Previous research has indicated that influenza virus co-infection with *S. aureus* in children is associated with a 9-fold increase in mortality (Kalil and Thomas, 2019), highlighting the need for clinical vigilance regarding this co-infection. Notably, MSSA, like MRSA, is also a significant cause of NP. Roberts et al. reported a fatal case of NP caused by co-infection with influenza virus and Panton-Valentine leucocidin (PVL)-positive CA-MSSA, emphasizing that antibiotic susceptibility does not equate to low virulence, and that CA-MSSA can induce a clinical course as fulminant as that caused by MRSA (Roberts et al., 2008). The present case, involving a life-threatening septic shock, NP, and *S. aureus* detected in blood by mNGS caused by co-infection with influenza A virus (H3N2) and CA-MSSA, is consistent with previous reports. Unfortunately, due to the limitations of our institutional laboratory facilities, genotyping and virulence factor detection of *S. aureus* could not be performed. Nevertheless, in conjunction with similar cases reported in the literature (Bai et al., 2020; Chen et al., 2024; Zheng et al., 2026), we speculate that the *S. aureus* in this case may have been PVL-positive.

NP resulting from co-infection with influenza virus and CA-MSSA is associated with a fulminant clinical course and high mortality rates (Profir et al., 2025; Soultan et al., 2025). Early and effective antimicrobial therapy is critical to improving clinical outcomes. During influenza season, empirical coverage against MRSA with vancomycin or linezolid should be considered, particularly when influenza patients present with signs of NP or sepsis, including hemoptysis, pleural effusion, ARDS, or leukopenia (Park et al., 2015). However, NP is frequently accompanied by tissue destruction and toxin-mediated pathological damage, and antibiotic therapy alone may be insufficient to control the disease. For NP caused by PVL-positive strains, in which toxin-mediated tissue injury is more severe, the use of agents that suppress bacterial toxin synthesis, such as LZD or clindamycin, is recommended (Fujisaki et al., 2014; Cuddihy et al., 2021; Profir et al., 2025). LZD exhibits favorable pulmonary tissue penetration and has been shown to suppress the production of PVL and other toxins, rendering it particularly valuable in the management of severe MSSA pneumonia (Cuddihy et al., 2021; Profir et al., 2025). Furthermore, in patients with *S. aureus* biofilm formation in the airways, LZD achieves higher concentrations within biofilms, thereby facilitating bacterial clearance (Fernández-Barat et al., 2019). In the present case, although the MSSA isolate was susceptible to vancomycin *in vitro*, clinical failure was evident after 9 days of vancomycin therapy. The switch to LZD was further supported by bronchoscopic evidence of extensive pseudomembranous formation, LZD’s superior pulmonary penetration and biofilm activity, and the need for toxin suppression—particularly given that inducible clindamycin resistance precluded the use of clindamycin as an alternative.

ECMO may serve as a bridging strategy to gain time for antimicrobial therapy; however, the ultimate prognosis remains dependent on early effective antibiotic treatment and comprehensive management of toxin-mediated injury (Criss et al., 2018; Cuddihy et al., 2021; Ding et al., 2025; Wang et al., 2026). The successful outcome in this case was attributable to early ECMO support and precise antimicrobial therapy, even though the patient subsequently developed mixed infections with multiple multidrug-resistant organisms.

The incidence of LILA in a multicenter study was approximately 10.7%, with a mean onset at day 4 (Naseralallah et al., 2026). Reports in children are rare (Ozkaya-Parlakay et al., 2014; Smolka et al., 2020; Asai et al., 2022; Ghandour et al., 2023; Song et al., 2024); however, a Turkish study reported rates of linezolid-associated hyperlactatemia (32%) and LILA (16%) in children (Ozkaya-Parlakay et al., 2014), both higher than in adults (Naseralallah et al., 2026). In our patient, LZD was administered at 10 mg/kg/dose intravenously every 8 hours (patient weight: 30 kg), with normal renal and hepatic function throughout therapy. The patient’s initial septic shock and hyperlactatemia resolved with antibiotics, vasopressors, and ECMO. However, on day 5 of LZD, hyperlactatemia and frequent vomiting recurred without identifiable gastrointestinal pathology. By day 15, lactate reached 9.4 mmol/L, prompting a diagnosis of lactic acidosis. No other causes (anemia, hypoxia, or hypoperfusion) were identified. Rapid normalization of lactate within 3 days of LZD cessation, supported by prior reports of LILA (Ozkaya-Parlakay et al., 2014; Smolka et al., 2020; Asai et al., 2022; Song et al., 2024), strongly suggested a causal relationship, with a Naranjo score of 7 indicating probable association (Naranjo et al., 1981). In our patient, the Naranjo score was calculated as follows: (1) appearance of hyperlactatemia after LZD initiation (+2); (2) resolution within 3 days of drug discontinuation (+1); (3) exclusion of alternative causes including tissue hypoperfusion, hypoxia, anemia, and hepatic/renal dysfunction (+2); (4) previous literature supporting this association (+1); and (5) objective evidence from serial lactate measurements (+1), yielding a total of 7 (probable).

Despite its relatively low incidence, LILA carries a high case-fatality rate of 25.5% (Mao et al., 2018). Accordingly, it is recommended that clinicians closely monitor serum lactate levels throughout the course of linezolid treatment to enable prompt identification of this life-threatening complication. In this case, hyperlactatemia was first observed on day 5 of LZD therapy; however, the diagnosis of LILA was not recognized until day 15 of treatment. This indicates insufficient awareness of LILA among pediatric clinicians and highlights the importance of improving knowledge of this uncommon adverse effect to minimize missed diagnoses and delayed recognition.

## 4 Conclusion

In conclusion, during influenza seasons, clinicians should maintain high suspicion for *S. aureus* co-infection in children with rapidly progressive pneumonia, leukopenia, hemoptysis, or septic shock, and initiate early empirical coverage even for MSSA. Concurrently, vigilant lactate surveillance is essential to detect LILA promptly and prevent excess mortality. Multidisciplinary intensive care, timely antimicrobial therapy, and adverse effect monitoring are key to improving outcomes in such devastating infections.

## Data Availability

The original contributions presented in the study are included in the article/supplementary material. Further inquiries can be directed to the corresponding author.
